# Comparison of the Intestinal Bacterial Communities between Captive and Semi-Free-Range Red-Crowned Cranes (*Grus japonensis*) before Reintroduction in Zhalong National Nature Reserve, China

**DOI:** 10.3390/ani14010003

**Published:** 2023-12-19

**Authors:** Yining Wu, Huan Wang, Zhongyan Gao, He Wang, Hongfei Zou

**Affiliations:** 1 College of Wildlife and Protected Area, Northeast Forestry University, Harbin 150040, China; wuyiningnefu@126.com (Y.W.); dl.wanghuan@foxmail.com (H.W.); wanghenefu@126.com (H.W.); 2 Management Bureau of Heilongjiang Zhalong National Reserve, Qiqihar 161005, China; gaozhongyan0451@163.com

**Keywords:** red-crowned crane, gut microbiota, high-throughput sequencing technology, reintroduction, Zhalong Nature Reserve

## Abstract

**Simple Summary:**

Gut microbiota is diverse and complex and is a key environmental factor affecting the immune homeostasis of the host. The results showed significant differences in gut microbiota community structures between semi-free-range and captive red-crowned cranes. Comparing the differences in gut microbiota function and composition of captive and semi-free-range red-crowned cranes is critical for conservation management and policy making, as well as the projects focusing on the release of captive red-crowned cranes into the wild.

**Abstract:**

The wild populations of red-crowned cranes (*Grus japonensis*) in west China are gradually decreasing, necessitating the optimization of reintroduction measures. This study used 16S rRNA high-throughput sequencing technology to compare the gut microbiota communities of cranes living in two modes (captive and semi-free-range) before their reintroduction in Zhalong National Nature Reserve, Heilongjiang Province, China. The results showed that Proteobacteria (74.39%) and Firmicutes (25.29%) were the dominant gut bacterial phyla inhabiting these cranes. Significant differences were found in the gut microbiota community composition between semi-free-range and captive cranes (*p* < 0.01). *Psychrobacter*, *Sporosarcina*, and *Lactococcus* were significantly enriched in captive cranes (*p* < 0.05), while *Pseudomonadaceae_Pseudomonas*, *Pantoea*, *Lysobacter*, and *Enterobacteriaceae_Pseudomonas* were more abundant in semi-free-range cranes (*p* < 0.05). The functions and community structure of gut microbiota were affected by feeding patterns (*p* < 0.05). The metabolic pathways of ethylbenzene degradation, PPAR signaling pathway, betalain biosynthesis, systemic lupus erythematosus, and shigellosis were up-regulated in semi-free-range cranes *(p* < 0.05).

## 1. Introduction

Through long-term co-evolution with their host, intestinal microorganisms have become an “organ” in and of themselves [1] and perform various functions, including substance synthesis and metabolism [2], provision of biological barriers [3], immune regulation [2], and host defense [4,5]. Intestinal microorganisms are affected by many factors, including host species, dietary strategies, and life history characteristics, such as migration behavior [6,7,8].

The red-crowned crane (*Grus japonensis*) is an indicator species of the wetland environment [9]. However, due to the massive loss and deterioration of their habitat and the disturbance caused by increasing human activity, the number of wild populations of red-crowned cranes in west China is gradually decreasing [10]. Captive populations are the principal source for endangered wild animal populations that can be reintroduced into natural or reconstructed habitats [11]. Based on the “peer cluster” behavior of cranes, Zhalong National Nature Reserve in Heilongjiang has adopted two modes for reintroducing red-crowned cranes. The wild population is augmented by natural escapes of captive red-crowned cranes during flight training and by migration of natural propagation offspring of semi-free-range cranes. By the end of 2014, nearly 200 red-crowned cranes had been fully reintroduced into the Zhalong reserve. Nonetheless, the reintroduction of artificially bred populations of endangered wild animals is a complex and long-term process. Field training is conducive to the rebuilding of the gut microbiota communities of animal populations raised via artificially assisted breeding. It is also beneficial for increasing their survival rate after their release into the wild.

Feeding patterns are important factors for such reintroduction efforts. Differences in gut microbiota have been found between captive and wild or free-ranging birds, such as the Western capercaillie (*Tetrao urogallus*) [12], blue-fronted Amazon parrot (*Amazona aestiva*) [13], Chinese monals (*Lophophorus lhuysii*) [14], oriental white storks (*Ciconia boyciana*) [15], and bar-headed geese (*Anser indicus*) [16]. Zhalong National Nature Reserve is located in the middle of the East Asia–Australasia bird migration corridor, which is dominated by reed swamp. Many rare species of waterfowl, mostly dominated by cranes, especially red-crowned cranes and white-naped cranes, breed and live here. The unique geographical location and typical ecosystem of Zhalong make it an important breeding ground and habitat for birds in Northeast China. Zhalong National Nature Reserve, therefore, plays a crucial role in crane protection and wetland protection.

The fecal microbiota does not fully reflect the gut microbiota, and the efficacy of fecal sampling in accurately representing the gut microbiota of birds is limited. However, due to its non-invasive nature, fecal sampling can serve as a simple and non-invasive method for endangered species. For this study, fresh fecal matter was collected from semi-free-range and captive red-crowned cranes over the same period. The semi-free-range and captive cranes were fed the same food in the Zhalong nature reserve during the sampling period. The food consisted of crucian carp, corn, and cabbage, preserved via frozen storage. In addition to artificial feeding, semi-free-range cranes could freely feed on wetland aquatic plants and animals, such as reed, loach, and dragon louse. The 16S rDNA high-throughput sequencing method was used to explore how reintroduction affects red-crowned crane populations. The following hypotheses were tested: (1) there are pronounced differences in the gut microbiota community compositions due to different feeding patterns. (2) The composition and function of gut microbiota communities differ based on feeding patterns. The effects of feeding patterns on the gut microbiota of cranes were explored before their reintroduction into the Zhalong Nature Reserve. The results provide a theoretical basis for the research of red-crowned crane reintroduction measures that may also be important for other species.

## 2. Materials and Methods

### 2.1. Ethics Statement

Fecal samples were obtained using a non-invasive method, which was approved by the Heilongjiang Zhalong National Nature Reserve, to avoid unnecessary human interference with red-crowned cranes during the sampling process.

### 2.2. Site Selection and Sample Collection

The research area was Zhalong National Nature Reserve, located in the west of Heilongjiang Province, southeast of Qiqihar City (N46°52′–47°32′, E123°47′–124°37′), with a total area of 2.1 × 105 hm^2^. The area has a continental semi-arid monsoon climate, with an average annual precipitation of 420 mm, an average annual temperature of 3.9 °C, and southerly winds during the summer, with an average annual wind speed of 3.5 m/s. The average annual sunshine time is 2864 h, and the average annual frost-free period is 128 d [17].

Sixteen fresh fecal (non-invasive) samples of red-crowned cranes were collected in October 2020, including eight samples of semi-free-range cranes and eight samples of captive cranes. Approximately 1 g of fresh fecal samples was immediately collected in a 1.5 mL sterile centrifuge tube after defecation near their foraging place, to avoid fecal substances that have come in contact with the ground. The banding number of semi-free-range cranes was recorded. For captive cranes, fresh fecal samples were randomly collected from their cage with the assistance of staff. Feces were temporarily stored in an incubator on ice during the sampling process and were quickly transported back to the laboratory after sampling for storage at −80 °C.

### 2.3. Sample Pretreatment

The fecal samples were subjected to UV spectrophotometry detection using a Nanodrop NC200 (Thermo Scientific, Waltham, MA, USA) and later uniformly diluted to 20 ng/μL for (Polymerase Chain Reaction, PCR) amplification of the bacterial DNA. The hypervariable bacterial region V3V4 (a) was amplified using the forward primer ACTCCTACGGGAGGCAGCA and reverse primer GGACTACHVGGGTWTCTAAT. The reaction volume was 25 μL and contained 5 μL of 5 × reaction buffer, 5 μL of 5 × GC buffer, 2 μL of (deoxy-ribonucleoside triphosphate, dNTP) (2.5 mM), 1 μL forward and reverse primers (10 μM) each, 2 μL of DNA template, 8.75 μL of ddH_2_O, and 0.25 μL of Q5 DNA polymerase. The PCR reaction conditions were as follows: initial denaturation at 98 °C for 2 min, followed by 25–30 cycles of denaturation at 98 °C for 15 s, annealing at 55 °C for 30 s, and extension at 72 °C for 30 s, with a final extension at 72 °C for 5 min and the holding stage at 10 °C. The target bands were detected by agarose gel electrophoresis, followed by sequencing.

### 2.4. 16S rRNA Sequencing and Processing of Sequence Data

The DNA of the bacterial community was sequenced on the Illumina platform (USA). DADA2 [18] of QIIME2 (April 2019) software was used for primer removal, mass filtration, denoise, splicing, and chimerism, and the obtained amplicon sequence variants were searched against the Greengenes database [19] for the classification and annotation of species using the “classify-sklearn” function [20] of QIIME2. Since all samples should have the same sequencing depth before analysis, a certain number of sequences were randomly selected from each specimen, using rarefaction to predict ASVs and relative abundance at the same sequencing depth [21]. The leveling depth was set to 95% of the minimum sample sequence size using the “qiime feature-table rarefy” function of QIIME2 software. The 16S rRNA gene sequences were submitted to the NCBI’s sequence read archive (SRA) under the accession number PRJNA832609.

### 2.5. Statistical Analysis

The “qiime taxa barplot” command of QIIME2 (April 2019) was used to analyze the species composition of the bacterial community. The data were exported to the characteristic table after removing the “singleton” to visualize the composition distribution of each sample at the six classification levels: phylum, class, order, family, genus, and species. Alpha and Beta diversity indices were used to characterize species diversity within and between habitats, respectively, and to comprehensively evaluate the total species diversity. Chao1 and observed species indices were employed to indicate the richness, while Shannon and Simpson’s indices were employed to characterize diversity. Beta diversity analysis was conducted through principal coordinates analysis (PCoA). Based on the Bray–Curtis distance matrix, differences between groups were analyzed via permutational multivariate analysis of variance (PERMANOVA) using the Python package “scikit-bio”, with the replacement inspection time set to 999. Linear discriminant analysis effect size (LEfSe) analysis was employed to conduct difference analysis at all classification levels. LEfSe enabled the identification of robust differential species between groups, and these species were considered biomarkers. Finally, based on the KEGG database, the functional potential of the 16S rRNA gene sequences was predicted using the Picrust2 software. The packages of QIIME2 (April 2019), R, Python, and Picrust2 software were used for the analysis.

## 3. Results

### 3.1. Gut Microbial Community Composition of Captive and Semi-Free-Range Red-Crowned Cranes

**Figure 1:**
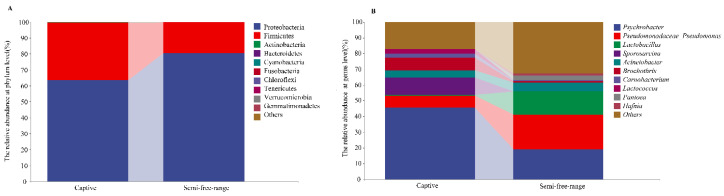
Relative abundances of the dominant bacteria in semi-free-range and captive red-crowned cranes. (**A**) dominant bacterial phyla, (**B**) dominant bacterial genera.

Proteobacteria and Firmicutes dominated the gut microbiota community of red-crowned cranes (Figure 1A, Table S1), and members of Proteobacteria (74.39%) were more abundant than Firmicutes (25.29%). According to the analysis of variance (ANOVA) results (Table 1), no significant difference in the abundance of the dominant phyla of the gut microbiota was found between semi-free-range and captive cranes (*p* > 0.05).

In the Zhalong Nature Reserve, the gut bacterial genera of red-crowned crane was greater than 1% (Figure 1B, Table S2) and included *Psychrobacter* (32.34%), *Pseudomonadaceae_Pseudomonas* (14.9%), *Lactobacillus* (7.61%), *Sporosarcina* (5.50%), *Acinetobacter* (5.01%), *Brochothrix* (4.41%), *Carnobacterium* (1.61%), *Lactococcus* (1.56%), and *Pantoea* (1.40%). Significant differences were found in the abundance of certain bacterial genera between semi-free-range and captive cranes. The abundance of *Psychrobacter* was higher in the captive group than in the semi-free-range group (*p* < 0.01), while the abundance of *Pseudomonadaceae_Pseudomonas* was higher in the semi-free-range group than in the captive group (*p* < 0.05), and members of *Sporosarcina* were mainly distributed in the captive group (*p* < 0.05). *Pseudomonadaceae_Pseudomonas* had the highest abundance in the semi-free-range group, while *Psychrobacter* had the highest abundance in the captive group.

**Figure 2:**
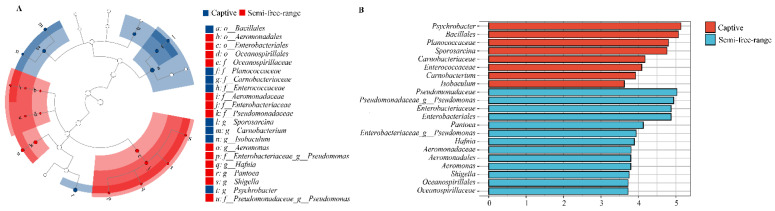
Linear discriminant analysis effect size (LEfSe) analysis (**A**) and Plot from LEfSe analysis (**B**) of intestinal bacteria in semi-free-range and captive red-crowned cranes.

According to the LEfse results, the gut microbial composition exhibited substantial differences between captive and semi-free-range red-crowned cranes (Figure 2A). The LDA scores of microbial taxa, with significant differences between captive and semi-free-range red-crowned cranes, are shown in Figure 2B. The bacteria with significantly higher abundance values in the captive red-crowned cranes than in the semi-free-range group were *Sychrobacter*, *Bacillales*, *Planococcaceae*, *Sporosarcina*, *Carnobacteriaceae*, *Enterococcaceae*, *Carnobacterium*, and *Isobaculum*. However, the semi-free-range red-crowned cranes had more unique flora compared with captive groups, including *Pseudomonadaceae*, *Enterobacteriaceae*, *Enterobacteriales*, *Pantoea*, *Pseudomonas*, *Hafnia*, *Aeromonadaceae*, *Aeromonadales*, *Aeromonas*, *Shigella*, *Oceanospirillales*, and *Oceanospirillaceae* (LDA > 2.0; *p* < 0.05).

### 3.2. Analysis of the Diversity of the Gut Microbiota of Captive and Semi-Free-Range Red-Crowned Cranes

**Figure 3:**
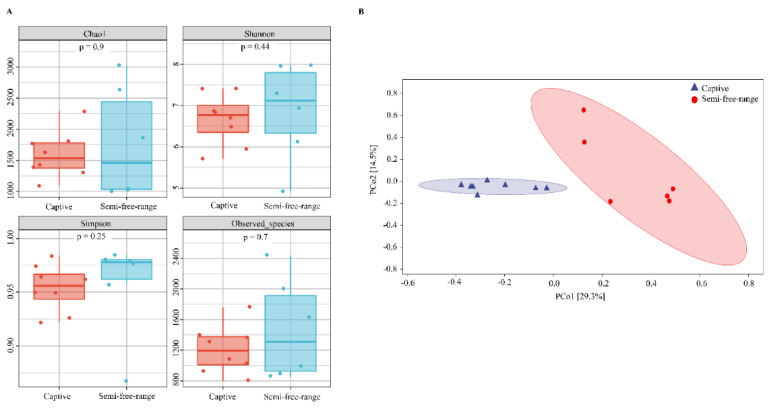
Alpha diversity index analysis (**A**) and PCoA analysis (**B**) of intestinal bacteria in semi-free-range red-crowned cranes at different genders.

The smoothness of the rarefaction curve reflects the influence of sequencing depth for the observation of sample diversity. The richness of bacterial species in the intestinal tract of cranes in the semi-free-range group was higher than that in the captive group at the same sequencing depth (Figure S1). However, no significant difference was found in Alpha diversity between semi-free-range and captive cranes. Moreover, there was no significant difference in the richness, diversity, and evenness of the gut microbiota community between both feeding patterns (*p* > 0.05) (Figure 3A). The Beta diversity of gut microbiota communities of semi-free-range and captive cranes in Zhalong Nature Reserve was explored through PCoA analysis. The results showed the differences in species composition between the sample groups. As shown in Figure 3B, the captive and semi-free-range samples were clearly separated, indicating that the compositions of gut microbiota communities of captive and semi-free-range cranes were significantly different.

### 3.3. Functional Potential Prediction of the Gut Microbiota of Captive and Semi-Free-Range Red-Crowned Cranes

**Figure 4:**
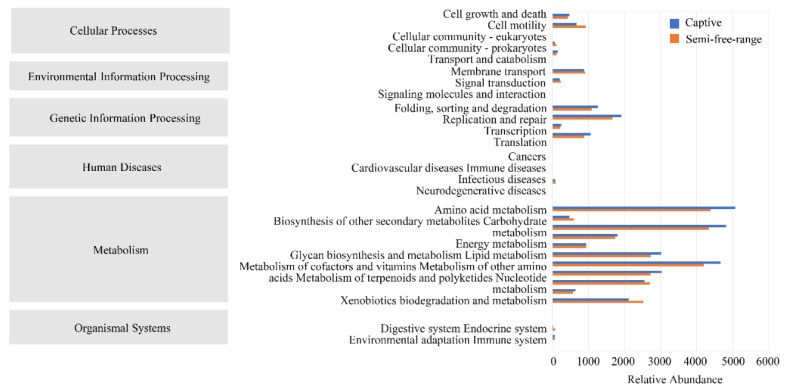
Metabolic pathway statistics of intestinal bacteria of captive and semi-free-range red-crowned cranes.

The functional potential of the gut microbiota of cranes in both the semi-free-range and captive groups was predicted based on the KEGG database (Figure 4). The enrichment analysis of functional pathways showed that metabolism is the most important function of the gut microbiota of the red-crowned crane. The specific second-level pathways of metabolism included amino acid metabolism, biosynthesis of other secondary metabolites, carbohydrate metabolism, energy metabolism, lipid metabolism, metabolism of cofactors and vitamins, metabolism of other amino acids, metabolism of terpenoids and polyketides, nucleotide metabolism, and xenobiotics biodegradation and metabolism.

According to the ANOVA results for the relative abundance of metabolic pathways in the second level of KEGG (Table S3), four metabolic pathways had higher relative abundances in the semi-free range than in the captive group. These were cellular community-prokaryotes (*p* < 0.01), endocrine system (*p* < 0.05), cardiovascular diseases (*p* < 0.01), and biosynthesis of other secondary metabolites (*p* < 0.01). There were also four metabolic pathways with higher relative abundance in the captive group, including transport and catabolism (*p* < 0.05), folding, sorting and degradation (*p* < 0.05), amino acid metabolism (*p* < 0.01), and metabolism of other amino acids (*p* < 0.05).

Five metabolic pathways, including KO00642 (ethylbenzene degradation), KO03320 (PPAR signaling pathway), KO00965 (betalain biosynthesis), KO05322 (systemic lupus erythematosus), and KO05131 (shigellosis), were significantly up-regulated in semi-free-range cranes compared to captive cranes, based on the third level of KEGG (*p* < 0.05) (Figure S2).

## 4. Discussion

This is the first study to present a comparative analysis of the gut microbiota composition between captive and semi-free-range red-crowned cranes at Zhalong National Nature Reserve in Heilongjiang Province, China. High-throughput sequencing technologies were employed to determine the differences between gut microbiota between captive and semi-free-range red-crowned cranes under different feeding patterns. 

Proteobacteria and Firmicutes were the dominant phyla in captive and semi-free-range red-crowned cranes, consistent with the results of previous studies on the gut microbiota of omnivorous birds such as chickens and hornbills [22,23,24,25]. Moreover, these two phyla are generally highly prevalent in omnivorous birds, such as vultures [26] and penguins [27], and herbivorous birds, such as geese and parrots [28,29]. Proteobacteria and Firmicutes play an essential role in the physiological and biochemical functions of certain birds, such as regulation of the short digestive tract, the supplementation of high energy demand during flight, and the determination of reproductive patterns (e.g., oviposition) [30]. However, a previous study found that the dominant bacteria in the fecal samples of red-crowned cranes in Yancheng, China, were Firmicutes, Proteobacteria, and Fusobacteria [31]. These differences may be due to several reasons. First, the variation in the relative abundance of dominant bacteria in birds may be affected by diet [32]. Distinct habitats provide different food compositions for red-crowned cranes, and since the sampling time was not identical between studies (i.e., different seasons), the composition of foods birds may have encountered during feeding might have been different. Secondly, the sample sources for the two studies were different. Research has shown that the location of sampling sites mainly reflects the composition of the microbial community, and the geographical distance is positively correlated with differences in microbial flora. Local animals and plants, light cycles, available food, and climatic conditions may affect the microbial flora, and the effect may be different between different regions [31]. Finally, differences in analytical methods might have also caused the differences in results [33].

The higher abundance of Proteobacteria in cranes was related to the cold climate of the Zhalong reserve. Studies have shown that the increase in Proteobacteria is mainly related to energy accumulation [34,35], and their abundance is higher in host animals living in cold climates for a prolonged time [36]. The temperature of the living environment of semi-free-range cranes is lower than that for captive cranes, and cranes need plentiful energy to avoid danger, breed, and brood independently. Therefore, semi-free-range cranes evolved to have a higher abundance of Proteobacteria. Firmicutes are mainly involved in the decomposition of carbohydrates and polysaccharides, and the decomposed products can be absorbed by the host [37,38]. The higher abundance of Firmicutes in captive cranes may be related to the large amount of corn with high starch content in their diets [39,40,41].

*Psychrobacter*, *Sporosarcina* [42], and *Brochothrix* [43] are distributed throughout the natural environment, suggesting that horizontal transmission affects the structure of the gut microbiota of animals. Also, it could be that the fecal samples were polluted with soil during sampling. While these genera were more abundant in captive cranes, their effect on the host remains unclear [44,45]. In addition, it has been shown that leopard seals (*Hydrurga leptonyx*) have a high abundance of operational taxonomic units from Proteobacteria, especially *Psychrobacter* [46]. Moreover, these bacterial species have been detected in Antarctic krill (*Euphausia superba*), Adélie penguins (*Pygoscelis adeliae*) and gentoo penguins (*P. Papua*) [47,48], which are common prey for leopard seals [49]. *Psychrobacter* are renowned for inhabiting low-temperature and high-salt environments, such as sea ice, permafrost, and frozen food [50]. The diet of cranes in Zhalong Reserve was occasionally supplemented with frozen crucian carp during the sampling process. Thus, the high abundance of *Psychrobacter* might have been due to frozen crucian carp supplementation. *Psychrobacter* and *Lactococcus* were enriched in the intestines of captive cranes, while *Pseudomonadaceae_Pseudomonas* was enriched in the intestines of semi-free-range cranes. Both *Psychrobacter* and *Lactococcus* positively correlated with the acetic acid, propionic acid, and butyric acid contents, indicating that they can increase the contents of intestinal short-chain fatty acids, reduce the pH of the intestinal environment, and inhibit the growth of harmful gut microbiota [51]. *Pseudomonas* was positively correlated with isobutyric acid, which can promote the growth of fibrinolytic bacteria and increase the activity of plasmin. This might have been due to the higher amount of wetland plants in the diet of semi-free range cranes [52].

Diversity analysis showed that the Alpha diversity of gut microbiota between captive and semi-free-range cranes was not significantly different (*p* > 0.05), probably because the host species were identical, and their food was mainly consistent during the sampling period [39]. In October, the volume of available plants for semi-free-range cranes in Zhalong reserve decreased. The foods of the two groups of red-crowned cranes were mainly corn and crucian carp, and, occasionally, Chinese cabbage. However, the rarefaction curve showed that the species richness of the semi-free-range group was higher than that of the captive group, probably due to the diversity of food sources of semi-free-range cranes. Semi-free-range cranes were allowed to forage freely and supplement their diet with artificial feed daily. In spring and summer, semi-free-range cranes could freely feed on animal foods such as fish, shrimp, and plants with high crude protein and crude fat contents, such as corn, reeds, *Carex scabrifolia*, and *Suaeda*, that are available in the Zhalong wetland. Furthermore, dietary supplementation with a moderate amount of cellulose can increase the quantity of gut microbiota [53]. Gut microbiota form a dynamic ecosystem that is susceptible to many factors, including the dietary habits, lifestyle, age, and genotype of the host, of which diet is one of the principal determinants [54]. Although there is limited knowledge on the extent to which the dietary fluctuations affected the host gut microbiota [16], PERMANOVA analysis showed significant differences in the species composition of gut microbiota between captive and semi-free-range cranes (*p* = 0.001). The communities of gut microbiota of captive and semi-free-range cranes differed because of their different living environments. Metabolic rates, physical conditions, hormone secretion, and other functions are altered when animals are in captivity [55,56], thus affecting gut microbiota [57]. Also, close contact with many other animals in captivity and sometimes with human caretakers may increase the chances of bacterial community transmission [58,59]. Semi-free-range cranes live near their nest site as a family unit. When food is plentiful during spring and summer, they can forage on diverse food sources. Microbes from the environment enter and colonize the digestive system of the red-crowned crane through the diet [60]. For example, some semi-free-range cranes dipped crucian carps in puddles near their nests before eating them, increasing the chances of some of the microbes from this environment entering their digestive tracts.

The results showed that the primary function of gut microbiota is metabolism. Amino acid metabolism, carbohydrate metabolism, and metabolism of cofactors and vitamins were highly enriched. These pathways may play an essential role in absorbing and assimilating energy. Cellular community—prokaryotes, endocrine system, cardiovascular diseases, and biosynthesis of other secondary metabolites were more highly enriched in semi-free-range red-crowned cranes than in captive cranes. This may be because semi-free-range cranes live as a family unit, and therefore, certain behaviors associated with occupying and protecting the nest might affect their endocrine system [61]. Semi-free-range cranes have a higher risk of cardiovascular diseases as they mature. The two metabolic pathways of the cellular community—prokaryotes and biosynthesis of other secondary metabolites were enriched in semi-free-range cranes. Since these two pathways improve digestion efficiency and assimilation [62] and provide more energy and nutrition, they likely play a critical role in enhancing the physique of cranes and helping them cope with a complex natural environment. Four metabolic pathways (amino acid metabolism, metabolism of other amino acids, folding, sorting and degradation, and transport and catabolism) were more abundant in captive cranes, probably due to the existence of juveniles in the captive group. These pathways are conducive to the absorption of nutrients and promote the growth and development of cranes. In the third level metabolic pathway, KO00642 (ethylbenzene degradation), KO03320 (PPAR signaling pathway), KO00965 (betalain biosynthesis), KO05322 (systemic lupus erythematosus), and KO05131 (shigellosis) were significantly up-regulated in semi-free-range cranes. The high abundance of these pathways is related to the living environment of semi-free-range cranes. The complex natural environment of semi-free-range cranes exposes them to more challenges than their captive counterparts. The high abundance of KO00642 is related to environmental pollutants [63], while that of KO00965 may be associated with the degradation of aromatic compounds [64] (Christinet et al., 2004). KO03320 plays a crucial role in regulating inflammation and glucose metabolism [65], which may be related to the metabolic pathways of KO05322 and KO05131. 

## 5. Conclusions

This study explored the effects of feeding patterns on the gut microbiota composition of the red-crowned crane in Zhalong Nature Reserve, Heilongjiang Province, China. The results showed significant differences in gut microbiota community structures between semi-free-range and captive red-crowned cranes (*p* < 0.01). *Psychrobacter* was significantly abundant in the intestine of captive cranes (*p* < 0.01), while *Pseudomonadaceae_Pseudomonas* was significantly increased in the gut microbiota community of semi-free-range cranes, probably due to cellulose decomposition (*p* < 0.05). The gut microbial function of the red-crowned cranes was affected by feeding patterns. Moreover, the metabolic pathways related to the living natural wetland environment, such as ethylbenzene degradation and PPAR signaling pathways, were significantly increased in the gut microbiota of semi-free-range cranes. The results presented in this paper provide a basis for understanding the gut microbiota community composition and potential bacterial function of red-crowned cranes in the Zhalong reserve.

## Figures and Tables

**Table 1:** One-way ANOVA of the relative abundance of dominant phyla of semi-free-range and captive red-crowned cranes.

	Feeding Patterns (Mean ± Standard Deviation)		*F*	*p*
	Captive (*n* = 8)	Semi-Free-Range (*n* = 8)		
Proteobacteria	0.64 ± 0.20	0.85 ± 0.33	2.567	0.131
Firmicutes	0.36 ± 0.19	0.14 ± 0.33	2.536	0.134
Actinobacteria	0.00 ± 0.00	0.00 ± 0.00	0.327	0.576
Bacteroidetes	0.00 ± 0.00	0.00 ± 0.00	0.040	0.845
Chloroflexi	0.00 ± 0.00	0.00 ± 0.00	3.517	0.082
Cyanobacteria	0.00 ± 0.00	0.00 ± 0.00	0.669	0.427
Fusobacteria	0.00 ± 0.00	0.00 ± 0.00	0.097	0.760
Verrucomicrobia	0.00 ± 0.00	0.00 ± 0.00	0.306	0.589
Tenericutes	0.00 ± 0.00	0.00 ± 0.00	0.126	0.728
Acidobacteria	0.00 ± 0.00	0.00 ± 0.00	3.594	0.079

## Data Availability

All 16S rRNA gene sequences obtained in the present study are available in the NCBI database under the bioproject number PRJNA832609.

## Supplementary Materials

The following supporting information can be downloaded at: https://www.mdpi.com/article/10.3390/ani14010003/s1. Figure S1: rarefaction curves of intestinal bacteria of semi-free-range and captive red-crowned cranes. Figure S2: analysis of metabolic pathway differences between semi-free-range and captive red-crowned cranes based on the third KEGG classification level. Table S1: relative abundances of the dominant bacteria at the phylum level in semi-free-range and captive red-crowned cranes. Table S2: relative abundances of the dominant bacteria at the genus level in semi-free-range and captive red-crowned cranes. Table S3: one-way ANOVA of the relative abundance of metabolic pathways based on the second KEGG classification level in semi-free-range and captive red-crowned cranes intestinal bacteria.
