# Supplementary material for: Comparison of the Intestinal Bacterial Communities between Captive and Semi-Free-Range Red-Crowned Cranes (Grus japonensis) before Reintroduction in Zhalong National Nature Reserve, China
Source: Animals (Basel). 2023 Dec 19;14(1):3. doi: 10.3390/ani14010003 (PMC10778468; doi:10.3390/ani14010003)
Supplement: Supplementary file 1 [file animals-14-00003-s001.zip › animals-2714768-Supplementary materials.pdf]

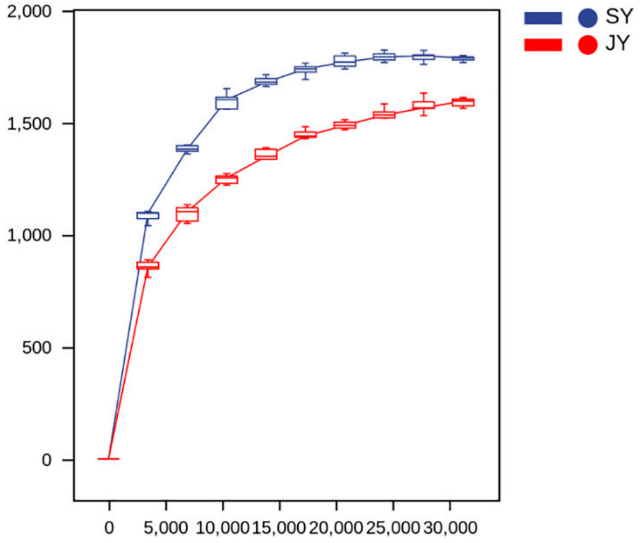

Fig. S1 Rarefaction curves of intestinal bacteria of semi-free-range and captive red-crowned cranes.

Note: SY, Red-crowned cranes in semi-free-range, JY, Red-crowned cranes in captivity.

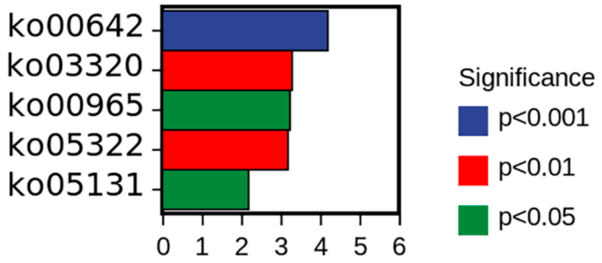

Fig. S2 Analysis of metabolic pathway differences between semi-free-range and captive red-crowned cranes based on the third KEGG classification level.

Note: The positive value of logFC(log2(fold change)) on the horizontal axis represents up-regulation in the upregulated group compared with the control group, while the negative value represents down-regulation. The vertical coordinates are different Pathway tags; Show the degree of significance in different colors. Upregulation group: SY, semi free-range red-crowned cranes, control group: JY, captive red-crowned cranes.

Table S1. Relative abundances of the dominant bacteria at the phylum level in semi-free-range and captive red-crowned cranes.

Phylum	Semi-free-range	Captive
Proteobacteria	63.51%	80.51%
Firmicutes	36.09%	19.28%
Actinobacteria	0.16%	0.09%
Bacteroidetes	0.02%	0.02%
Cyanobacteria	0.00%	0.01%
Fusobacteria	0.00%	0.01%
Chloroflexi	0.00%	0.00%

Tenericutes	0.00%	0.00%
Verrucomicrobia	0.00%	0.00%
Gemmatimonadetes	0.00%	0.00%
Others	0.21%	0.08%

Table S2. Relative abundances of the dominant bacteria at the genus level in semi-free-range and captive red-crowned cranes.

Genus	Semi-free-range	Captive
Psychrobacter	45.61%	19.08%
Pseudomonadaceae_Pseudomonas	7.70%	22.09%
Lactobacillus	0.46%	14.76%
Sporosarcina	10.99%	0.01%
Acinetobacter	4.42%	5.60%
Brochothrix	8.12%	0.71%
Carnobacterium	2.47%	0.76%
Lactococcus	3.05%	0.08%
Pantoea	0.00%	2.79%
Hafnia	0.00%	1.50%
Others	17.18%	32.62%

Table S1 One-way ANOVA of the relative abundance of metabolic pathways based on the second KEGG classification level in semi-free-range and captive red-crowned cranes intestinal bacteria.

	Feeding patterns (mean ± standard deviation)		*F*	*p*
	captive (*n*=8)	semi-free-range (*n*=8)		
Cell growth and death	476.19±44.16	425.57±82.12	2.358	0.147
Cell motility	665.14±339.80	923.26±413.24	1.862	0.194
Cellular community - eukaryotes	0.00±0.00	0.00±0.00	null	null
Cellular community - prokaryotes	66.23±26.48	113.99±21.79	15.520	0.001**
Transport and catabolism	154.27±30.87	117.91±29.06	5.886	0.029*
Membrane transport	882.71±131.50	911.05±74.92	0.280	0.605
Signal transduction	207.73±38.37	238.58±58.56	1.553	0.233
Signaling molecules and interaction	0.00±0.00	0.07±0.13	2.196	0.161
Folding, sorting and degradation	1261.35±141.29	1091.58±145.67	5.598	0.033*
Replication and repair	1917.30±219.06	1672.84±440.10	1.978	0.181
Transcription	245.34±42.31	219.30±29.04	2.059	0.173
Translation	1065.18±174.77	887.29±319.00	1.914	0.188
Metabolism of terpenoids and polyketides	2552.34±299.13	2702.97±108.20	1.794	0.202
Nucleotide metabolism	643.98±86.19	576.45±167.54	1.027	0.328

Xenobiotics biodegradation and metabolism	2118.51±592.62	2523.95±256.20	3.155	0.097
Digestive system	0.01±0.01	0.08±0.11	2.750	0.119
Endocrine system	30.32±35.53	87.43±47.37	7.442	0.016*
Environmental adaptation	68.09±6.39	64.64±5.24	1.400	0.256
Immune system	4.60±8.83	2.25±6.35	0.376	0.550
Cancers	1.55±4.39	0.00±0.00	1.000	0.334
Cardiovascular diseases	0.01±0.01	0.20±0.17	10.397	0.006**
Immune diseases	0.00±0.00	0.03±0.05	2.644	0.126
Infectious diseases	78.19±31.01	87.83±23.40	0.493	0.494
Neurodegenerative diseases	2.93±8.28	4.49±12.69	0.085	0.775
Amino acid metabolism	5074.07±240.78	4381.37±506.46	12.207	0.004**
Biosynthesis of other secondary metabolites	475.17±69.46	597.57±57.07	14.830	0.002**
Carbohydrate metabolism	4816.11±487.83	4339.79±407.82	4.490	0.052
Energy metabolism	1808.56±194.49	1745.41±116.09	0.622	0.444
Glycan biosynthesis and metabolism	946.33±140.65	943.99±76.20	0.002	0.968
Lipid metabolism	3021.38±253.91	2729.95±429.37	2.731	0.121
Metabolism of cofactors and vitamins	4666.45±437.09	4201.01±483.23	4.082	0.063
Metabolism of other amino acids	3029.60±201.67	2719.14±347.08	4.785	0.046*

*$p<0.05$ **$p<0.01$
