# Supplementary material for: Comparison of the Intestinal Bacterial Communities between Captive and Semi-Free-Range Red-Crowned Cranes (Grus japonensis) before Reintroduction in Zhalong National Nature Reserve, China
Source: Animals (Basel). 2023 Dec 19;14(1):3. doi: 10.3390/ani14010003 (PMC10778468; doi:10.3390/ani14010003)
Supplement: Supplementary file 1 [file animals-14-00003-s001.zip › Certification.pdf]

MogoEdit

CERTIFICATE OF ENGLISH EDITING

This is to certify that the manuscript entitled

Comparison of the intestinal bacterial communities between captive and semi-free-range red-crowned cranes (Grus japonen-sis) before reintroduction in Zhalong National Nature Reserve, China

commissioned to us has been carefully edited by a native English-speaking editor of MogoEdit, and the grammar, spelling, and punctuation have been verified and corrected, except the figure and table captions. Based on this review, we believe that the language in this paper meets academic journal requirements. Please contact us with any questions.

MOGOEDIT
MOGO INTERNET TECHNOLOGY CO., LTD.

Gang Zhang

Dr. Gang Zhang
Founder & CEO of MogoEdit

Date of Issue
December 1, 2023

Disclaimer: The changes in the document may be accepted or rejected by the authors in their sole discretion after our editing. However, MogoEdit is not responsible for revisions made to the document after our edit on **December 1, 2023**.

MogoEdit is a professional English editing company who provides English language editing, translation, and publication support services to individuals and corporate customers worldwide. As a company invested by the affiliate fund of Chinese Academy of Science, MogoEdit is one of the leading language editing service providers in China, whose clients come from more than 1000 universities and research institutes.
MogoEdit Website: http://www.mogoedit.com/
500+ native English editors: http://www.mogoedit.com/editors

Mogo
make the world flatter

Mogo Internet Technology Co., LTD.
No. 57, 3rd Keji Road, Xi'an 710075, PR China +86 02988317483 support@mogoedit.com
